# Supplementary material for: Detailed molecular and epigenetic characterization of the pig IPEC-J2 and chicken SL-29 cell lines
Source: iScience. 2023 Feb 20;26(3):106252. doi: 10.1016/j.isci.2023.106252 (PMC10018572; doi:10.1016/j.isci.2023.106252)
Supplement: Data S2. Complete homer output for identified motifs in Chicken SL-29, related to Tables 5 and 6 — Homer motif analysis results for histone modifications H3K4me1, H3K4me3, H3K27ac, enhancers, and ATAC-seq of chicken SL-29 cell line. Parameters for possible false positives is as mentioned earlier for S5. [file mmc3.zip › Data_S2/S6/Chicken_SL_29/peak_files_enhancers/homerResults/motif2.similar.html]

motif2

## Information for motif2

T
C
G
A
T
C
A
G
A
C
T
G
T
C
G
A
C
G
T
A
T
C
G
A
G
A
C
T
C
A
T
G
T
G
C
A
A
G
T
C
  
Reverse Opposite:  

T
C
A
G
A
C
G
T
G
T
A
C
C
T
G
A
A
G
C
T
C
G
A
T
A
G
C
T
T
G
A
C
A
G
T
C
A
G
C
T
  

|  |  |
| --- | --- |
| p-value: | 1e-91 |
| log p-value: | -2.104e+02 |
| Information Content per bp: | 1.722 |
| Number of Target Sequences with motif | 2392.0 |
| Percentage of Target Sequences with motif | 12.92% |
| Number of Background Sequences with motif | 2670.1 |
| Percentage of Background Sequences with motif | 8.47% |
| Average Position of motif in Targets | 149.8 +/- 82.7bp |
| Average Position of motif in Background | 149.2 +/- 85.9bp |
| Strand Bias (log2 ratio + to - strand density) | -0.0 |
| Multiplicity (# of sites on avg that occur together) | 1.08 |
| Motif File: | file (matrix) reverse opposite |

### Similar de novo motifs found

|  |  |  |  |  |  |  |  |
| --- | --- | --- | --- | --- | --- | --- | --- |
| Rank | Match Score | Redundant Motif | P-value | log P-value | % of Targets | % of Background | Motif file |
| 1 | 0.862 | G T A C G T C A A T C G A C T G C G T A C G T A C T G A A G C T | 1e-83 | -191.272408 | 17.07% | 12.17% | motif file (matrix) |
| 2 | 0.885 | C G T A C A T G G A C T T A G C C G T A G A C T A C G T A G C T T G A C G A T C A G C T A C T G A T G C | 1e-69 | -160.026148 | 0.33% | 0.01% | motif file (matrix) |
| 3 | 0.663 | T A C G A G C T A C G T A C G T G T A C G T A C C G A T A T C G A G T C G A T C | 1e-54 | -124.494026 | 21.81% | 17.35% | motif file (matrix) |
| 4 | 0.715 | G T A C A C T G A T C G C G T A C G T A A C T G A G C T A T C G | 1e-44 | -102.601462 | 4.20% | 2.44% | motif file (matrix) |
| 5 | 0.705 | C G T A C T A G G T A C C G A T A C G T A G T C A T G C A C G T | 1e-34 | -79.230649 | 16.97% | 13.76% | motif file (matrix) |
| 6 | 0.608 | A G T C C T G A A G T C A C G T A C G T A G T C A G T C A C T G A T C G A C G T | 1e-21 | -48.779461 | 0.21% | 0.03% | motif file (matrix) |
| 7 | 0.716 | C A T G A C T G T C G A C G T A T C A G G A C T A T C G T G C A A T G C T C A G A G C T T G C A T C G A | 1e-11 | -26.591390 | 0.08% | 0.01% | motif file (matrix) |
